# CCL2 and CCR2 regulate pain-related behaviour and early gene expression in post-traumatic murine osteoarthritis but contribute little to chondropathy

**DOI:** 10.1016/j.joca.2016.10.008

**Published:** 2017-03

**Authors:** J. Miotla Zarebska, A. Chanalaris, C. Driscoll, A. Burleigh, R.E. Miller, A.M. Malfait, B. Stott, T.L. Vincent

**Affiliations:** † Arthritis Research UK Centre for OA Pathogenesis, Kennedy Institute of Rheumatology, University of Oxford, Roosevelt Drive, OX3 7FY, UK; ‡ Department of Internal Medicine, Division of Rheumatology, Rush University Medical Center, Chicago, IL, UK

**Keywords:** CCL2, CCR2, Osteoarthritis, Animal model, Pain

## Abstract

**Objective:**

The role of inflammation in structural and symptomatic osteoarthritis (OA) remains unclear. One key mediator of inflammation is the chemokine CCL2, primarily responsible for attracting monocytes to sites of injury. We investigated the role of CCL2 and its receptor CCR2 in experimental OA.

**Design:**

OA was induced in 10 weeks old male wild type (WT), *Ccl2*^−/−^ and *Ccr2*^−/−^ mice, by destabilisation of the medial meniscus (DMM). RNA was extracted from whole joints at 6 h and 7 days post-surgery and examined by reverse transcription polymerase chain reaction (RT-PCR). Gene expression changes between naïve and DMM-operated mice were compared. Chondropathy scores, from mice at 8, 12, 16 and 20 weeks post DMM were calculated using modified Osteoarthritis Research Society International (OARSI) grading systems. Changes in hind paw weight distribution, as a measure of pain, were assessed by Linton incapacitance.

**Results:**

Absence of CCL2 strongly suppressed (>90%) selective inflammatory response genes in the joint 6 h post DMM, including arginase 1, prostaglandin synthase 2, nitric oxide synthase 2 and inhibin A. IL6, MMP3 and tissue inhibitor of metalloproteinase 1 were also significantly suppressed. Similar trends were also observed in the absence of CCR2. A lower average chondropathy score was observed in both *Ccl2*^*−/−*^ and *Ccr2*^−/−^ mice at 12, 16 and 20 weeks post DMM compared with WT mice, but this was only statistically significant at 20 weeks in *Ccr2*^−/−^ mice. Pain-related behaviour in *Ccl2*^−/−^ and *Ccr2*^−/−^ mice post DMM was delayed in onset.

**Conclusion:**

The CCL2/CCR2 axis plays an important role in the development of pain in murine OA, but contributes little to cartilage damage.

## Introduction

Although osteoarthritis (OA) is described as a non-inflammatory form of arthritis, it is frequently associated with low-grade synovitis and modestly elevated levels of inflammatory cytokines, systemically and within the synovial fluid^1^, ^2^. The role of inflammation in the structural and symptomatic course of disease is debated. Whilst cartilage degradation is induced readily *in vitro* following stimulation with inflammatory cytokines such as IL1, IL6 and TNF, these cytokines do not appear to have a major role *in vivo* in joint destabilisation models of OA, although not all studies agree^3^, ^4^, ^5^, ^6^, ^7^.

The chemokine CCL2, also referred to as monocyte chemotactic protein 1 (MCP1), is a key chemo-attractant molecule that binds to the cell surface leucocyte receptor CCR2. It recruits principally monocytes and to a lesser extent, memory T cells and dendritic cells to sites of inflammation. CCL2 is expressed in the synovial sublining cells of OA joints, and has been found in synovial fluid of OA knee joints^8^, ^9^ and following acute traumatic injury^10^, ^11^. *Ccl2* is strongly and rapidly (<6 h) induced in whole joints upon surgical joint destabilisation in the mouse^12^. *Ccl2* is also induced in rat cartilage upon surgical destabilisation^13^ and *in vitro* upon mechanical injury of cartilage^14^. The principal role of CCL2 in the joint may be to recruit leucocytes following joint injury. However, this role is unlikely to be the case in cartilage, an essentially avascular tissue, where several other mechanisms for chemokine action have been proposed^15^, ^16^.

CCR2 is expressed by sensory neurons, and ligation by CCL2 can directly excite nociceptive neurons, thereby contributing to pain^17^. Neuronal CCR2 is regulated transiently in the dorsal root ganglia 8 weeks following surgical joint destabilisation in animal models, where it is also associated with infiltration of macrophages, a potential source of algogenic molecules, and persistence of pain. In the absence of CCR2, there was transient distal mechanical allodynia (assessed by von Frey filaments) following destabilisation of the medial meniscus (DMM), but no development of motion-induced painful behaviour^18^. The Miller study also demonstrated that chondropathy scores in the *Ccr2*^−/−^ animals at 8 weeks post destabilisation were similar to those in wild type (WT) animals^18^. In another study, using incapacitance testing, where asymmetrical hind limb weight bearing is used as a surrogate measure of pain, we reported development of painful behaviour in WT animals between 10 and 12 weeks post joint destabilisation^19^. At this time, no regulation of Ccl2 or Ccr2 was detected in joint extracts or dorsal root ganglia of animals displaying pain-related behaviour. Nor was there up-regulation of other genes associated with an inflammatory response such as CD68, CD14, IL1, COX2 (^20^ and unpublished data).

Our hypothesis is that CCL2, acting through CCR2, contributes to structural and symptomatic disease in a murine model of OA. In the present study, we have performed a comprehensive analysis of the disease course in *Ccl2*^*−/−*^ and *Ccr2*^−/−^ mice at several time points post DMM. We show histological cartilage damage scores from the joints at these points, measure the acute inflammatory response in the joint over the first 7 days of surgery in knockout and WT animals, and examine pain-related behaviour responses in these groups over time.

## Methods

### Animals

Mice were kept in approved animal care facilities (individually ventilated cages and maintained under a 12-h light/dark cycle at an ambient temperature of 21°C) and were housed 4–6 per cage. The mice were fed a certified mouse diet (RM3; Special Diet Services) and water ad libitum. Animal experiments were performed following local ethics and statutory approval. *Ccr2*^*−/−*^ and *Ccl2*^*−/−*^ mice on a C57Bl/6J background were obtained from Jackson Laboratories (USA). Both colonies were maintained as homozygote breeding pairs. C57Bl/6J mice were used as controls and were purchased from Charles Rivers, UK.

### Surgical joint destabilization

Surgical joint destabilization was performed by DMM, in male mice at 10 weeks of age as previously described^21^ (total numbers of mice used: *Ccl2*^−/−^ *n* = 38, *Ccr2*^−/−^ *n* = 32, WT *n* > 30). All procedures had local (Imperial College then Oxford University) ethical approval. Some mice also underwent sham surgery (capsulotomy only; *n* = 4–6 at each time point). Briefly, mice were anaesthetized by inhalation of Isoflurane (4% induction and 1.5–2% maintenance) in 1 L/min oxygen. All animals received a subcutaneous injection of Vetergesic (Alstoe Animal Health Ltd) prior to surgery. The mice were fully mobile within 5 min following withdrawal of Isoflurane. The contralateral (left) knees were used as non-operated controls. Animals were sacrificed at 6 h, 7 days (for RNA extraction, see below) and 8, 12 and 20 weeks (for histology) after surgery. For histology, knee joints were fixed, decalcified, sectioned in the coronal plane and stained with Safranin O. Safranin O stained sections were scored according to a modified Osteoarthritis Research Society International (OARSI) score by two observers (blinded) and a summed score obtained. The summed score was the sum of the three highest total section scores for all four sections of the joint (minimum of eight sections per joint, 80 microns apart).

A separate set of experiments was performed independently and was approved by the Institutional Animal Care and Use Committee at Rush University Medical Center. Briefly, the anterior fat pad was dissected to expose the anterior medial meniscotibial ligament, which was severed. The knee was flushed with saline and the incision closed. Knee joints were collected 16 weeks after DMM from 12 WT and 11 *Ccr2*^−/−^ mice. These joints were processed and cartilage degeneration in the medial compartment was assessed histologically, as previously described^22^.

### RNA extraction and reverse transcription polymerase chain reaction (RT-PCR) for whole joints

Mice were sacrificed 6 h or 7 days after surgery, the skin and muscle were removed as previously described^12^ and the joints snap frozen in liquid N_2_ and stored at −80°C. The frozen joints were transferred to a prechilled biopulveriser in liquid N_2_ and pulverised to a fine powder. RNA from the pulverised tissue was extracted using 1 ml Trizol (ThermoFisher Scientific, UK) as previously described^12^, ^20^. 1 μg of total RNA was reverse transcribed using the High Capacity cDNA Reversion Transcription kit (ThermoFisher Scientific, UK) according to the manufacturer's protocol. The cDNA library was interrogated by qPCR on custom designed Taqman Low Density Array microfluidic cards (ThermoFisher Scientific, UK) on a ViiA7 thermocycler (ThermoFisher Scientific, UK). The Taqman probes used for each gene and the accession numbers for the analysed genes are listed in the Supplementary Data (Table S1). All TaqMan Low Density Arrays (TLDAs) were analysed together and C_t_ values extracted using the Expression Suit Software v 1.03 (ThermoFisher Scientific, UK). Fold changes for each gene were calculated using the ΔΔC_t_ method, employing the 40S ribosomal protein S18 (RPS18) gene as an endogenous control and using the average of the control ΔC_t_ values for each experiment as a normalizer.

### Pain-related behaviour assessments

Measurements of pain-related behaviour, as described previously were obtained twice weekly for the first week then at weekly intervals after DMM or sham surgery, by Linton incapacitance testing^19^, ^23^. The assessor was blinded with regard to treatment (sham or DMM) (but not genotype). Pain-related behaviour was judged to be meaningful when mean values dropped below 70% weight borne through the operated compared with non-operated joint. Statistical significance was determined by comparison of measurements with the sham-operated control.

### Statistical analysis

All groups of data were assessed for approximation to the Gaussian distribution using the D'Agostino and Pearson omnibus test of normality^24^. Distributions were considered to be Gaussian if the *P* value for the null hypothesis was greater than 0.05. When multiple comparisons between multiple end points were performed, the Bonferroni *post hoc* test was used to adjust for multiplicity^25^. GraphPad Prism version 6 was used for statistical analysis. To derive the sample size we performed power calculations based on previous data in WT mice 8 weeks post DMM^26^. Initial experiments were powered to detect a 50% change in chondropathy score (6 points), based on a standard deviation of 6.7, with 90% power and α set at 0.05. This gave 16 mice (eight in each group). Further animals were added subsequently to increase the power to detect a smaller difference between groups (see discussion for further details).

## Results

### Altered inflammatory responses in the joints of *Ccl2*^*−/−*^ and *Ccr2*^−/−^ mice following joint destabilisation

We first examined the functional effect of CCL2 or CCR2 deletion by comparing gene expression profiles of whole joints at 6 h and 7 days post surgical destabilisation. Several inflammatory response genes were strongly induced within 6 h of joint destabilisation (Table I). These included arginase 1 (Arg1), nitric oxide synthase (Nos2), *Ccl2*, IL6, Ptgs2 (Cox2), TNF-stimulated gene 6 (Tsg6) and hyaluronan synthases (Has) 1 and 2. A smaller number of these were still regulated 7 days post destabilisation; *Ccl2*, Nos2, Ptgs2 and Tsg6. Two macrophage/monocyte markers, Cd14 and Cd68 were examined. Cd14 was significantly raised at 6 h in WT joints. Cd68 was elevated but not significantly at this early time point.

Of the genes strongly regulated 6 h post destabilisation, a number of genes were suppressed in the *Ccl2*^−/−^ joints. Table II shows these results expressed as a ratio compared with WT levels. Those suppressed at 6 h by >90% included arginase 1, Ptgs2, Nos2 and inhibin beta A (Inhba). Other suppressed genes included Il6, Mmp3 and Timp1. For *Ccr2*^−/−^ joints, significantly suppressed genes at 6 h post DMM included Mmp3, Ptgs2, Arg1, Adamts4 and Inhba. For both *Ccl2*^−/−^ and *Ccr2*^−/−^ joints, Cd68 was significantly reduced compared with WT joint levels. There was no difference in Cd14 levels between WT and knockout joints. At 6 h, Arg2 was the only gene that was significantly higher in both *Ccl2*^*−/−*^ and *Ccr2*^−/−^ joints.

At 7 days post destabilisation fewer genes were regulated in WT joints. Nos2 and Ptgs2 were significantly suppressed in both *Ccl2*^*−/−*^ and *Ccr2*^*−/−*^ joints (Nos2 data not available for *Ccr2*^−/−^), whereas Has1/2, Mmp3 and Arg2 were super-induced in *Ccl2*^*−/−*^ joints. Mmp3 and Il1b were also super-induced in Ccr2^−/−^ joints. Taken together, the *Ccl2/Ccr2* knockout animals generally have reduced Nos2, arginase 1, Inhba, Mmp3 and Ptgs2 after joint destabilisation but also showed evidence of increased inflammatory markers especially at the later time point.

### Chondropathy scores in *Ccl2*^*−/−*^ and *Ccr2*^−/−^ mice are similar to WT controls

**Fig. 1:**
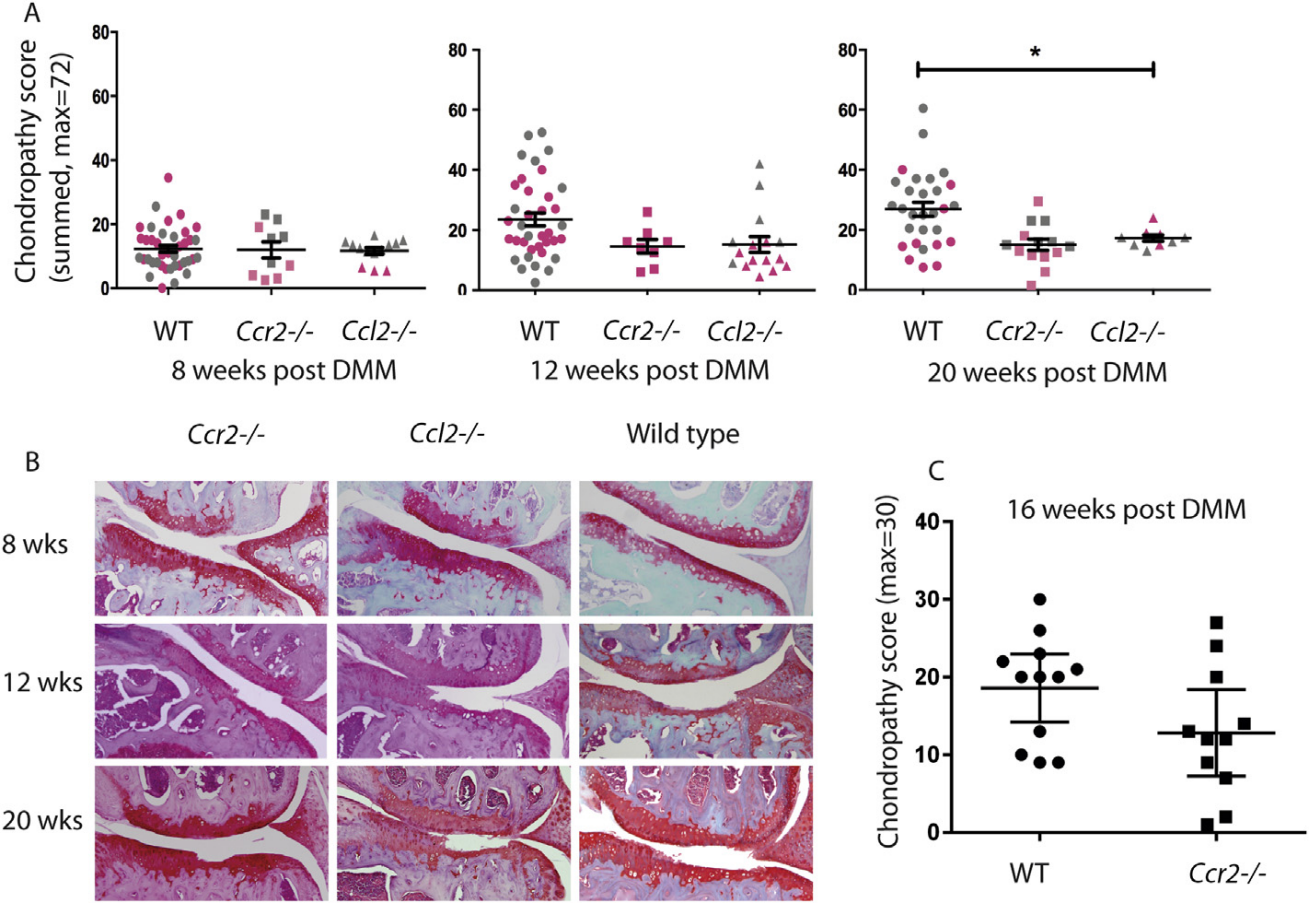
**Chondropathy scores are not substantially different in** WT**, *Ccl2***^***−/−***^**and *Ccr2***^**−/−**^**mice**. 10 weeks old, male WT (C57Bl/6J), *Ccl2*^*−/−*^ and *Ccr2*^*−/−*^ mice underwent DMM. Joints were harvested at 8, 12, 16 and 20 weeks post DMM for histological analysis and scored according to modified OARSI grading systems (each group using subtly different scores). Chondropathy scores (Vincent group) were pooled from several experiments performed over 4 years (A). Pink – experimental data acquired pre-2013; grey – experimental data acquired post-2013. Representative histology is shown (B). Histological scores using the same *Ccr2*^−/−^ strain but performed in a different laboratory (Malfait) are shown (C). Data was analysed by analysis of variance (ANOVA) with Bonferroni *post hoc* testing, *P ≤ 0.05. All other results non-significant.

To determine whether changes in inflammatory gene expression seen in *Ccl2*^*−/−*^ and *Ccr2*^*−/−*^ mice impacted on the degree of cartilage degradation following joint destabilisation, histological assessment of the joints of these mice was performed at 8, 12 and 20 weeks post DMM. The experiment was initially powered to detect a 50% change in disease, but numbers were subsequently added to increase the power to detect a 20% change in disease. Consequently, two sets of experiments were done involving different operators within the Vincent group, so the results are shown in two colours indicating the separate data sets [Fig. 1(B)]. Results show a significant difference (disease reduction; *P* < 0.05) in the *Ccl2*^−/−^ joints at 20 weeks post DMM compared with WT joints. *Ccl2*^*−/−*^ and *Ccr2*^−/−^ animals exhibited lower average scores at 12 weeks post DMM but these did not reach statistical significance. There were no significant differences in chondropathy scores at 8 weeks. Miller and Malfait corroborated the above results in a separate experiment from their lab (one surgeon); showing a non-significant trend towards decreased disease in *Ccr2*^−/−^ mice 16 weeks post DMM surgery [Fig. 1(C)].

There was no significant difference in histology scores in the sham-operated joints across genotypes (data not shown). Although there was a moderately wide distribution of scores in WT animals at all three time points post DMM there was neither a significant drift in disease severity over time, or change with operator (ANOVA, *P* > 0.05, for 8, 12 and 20 weeks post DMM in WT mice).

### Onset of pain-related behaviour following DMM surgery is significantly delayed in *Ccl2*^−/−^ and *Ccr2*^−/−^ mice

**Fig. 2:**
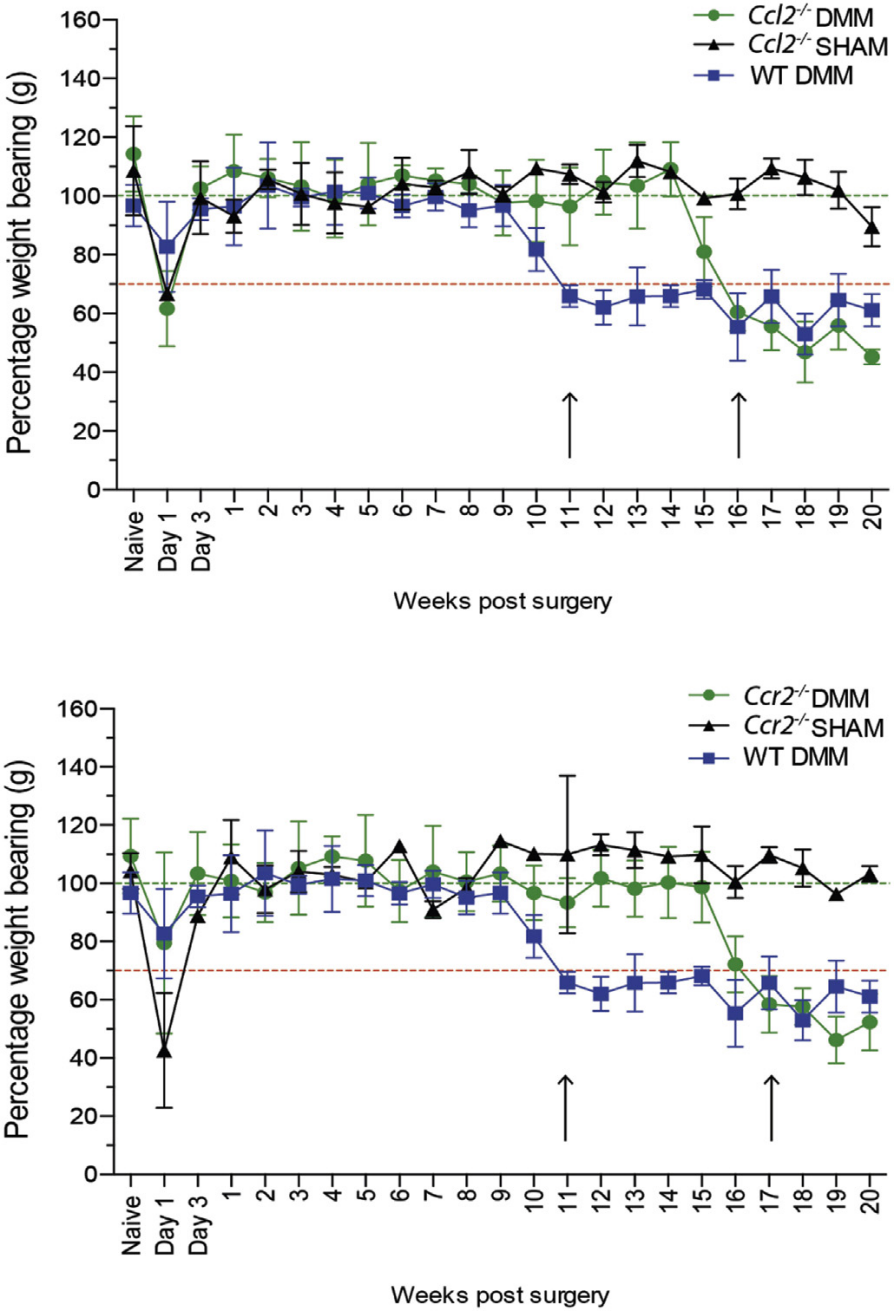
**Onset of pain-related behaviour is delayed in *Ccl2***^***−/−***^**and *Ccr2***^**−/−**^**mice post DMM**. 10 weeks old, male WT (C57Bl/6J), *Ccl2*^*−/−*^ and *Ccr2*^*−/−*^ mice underwent DMM or sham surgery. Pain-related behaviour was assessed twice weekly for the first week, then weekly by Linton Incapacitance testing. Statistical significance (ANOVA) was determined by comparing the difference between destabilised and sham-operated responses for *Ccl2*^*−/−*^ and *Ccr2*^−/−^ mice. Pain-related behaviour in WT mice is shown along side.

Pain-related behaviour was assessed twice weekly for the first week, then weekly by incapacitance testing, a measure of changes in hind paw weight distribution^18^. Male WT mice developed significant pain-related behaviour at 11 weeks post DMM (dropping below the 70% threshold of weight borne through the operated compared with non-operated side) [Fig. 2(A)]. Both *Ccl2*^−/−^ and *Ccr2*^*−/−*^ mice developed pain-related behaviour later; occurring at 16 and 17 weeks post DMM respectively [Fig. 2(A) and (B)]. Sham-operated *Ccl2*^*−/−*^ and *Ccr2*^*−/−*^ mice had normal weight bearing distribution beyond the immediate post-operative period. All mice displayed early post-operative pain-related behaviour (3–5 days) similarly.

## Discussion

The CCL2/CCR2 axis is the principal monocyte chemo-attractant pathway in humans and mice. Ccl2 is one of the most strongly regulated genes in whole joint extracts of mice after joint destabilisation and persists in the joint beyond 7 days. The RNA extracted from whole joint extracts is largely attributable to the subchondral bone, with relatively small amounts coming from synovium, articular cartilage and meniscus^27^. Nonetheless, Ccl2 is also strongly regulated in the articular cartilage in response to mechanical injury^12^, ^14^. Its roles in the joint are therefore likely to be varied; affecting both recruitment of monocytes and other leucocytes following destabilisation injury as well as having non-immunological roles. CCR2 is not significantly regulated upon joint injury but is expressed by many tissues of the joint including chondrocytes (Vincent unpublished data).

OA is not a classical inflammatory arthritis; inflammation is regarded as episodic and moderate, and it is debated whether synovitis contributes to the production of the pathogenic proteases (ADAMTS 5 and collagenases) that characterise disease. It is nonetheless the case that most patients with severe radiographic changes also show evidence of synovitis by magnetic resonance imaging (MRI), and synovitis probably contributes to pain in these individuals^28^, ^29^, ^30^. Our results show that deletion of CCL2 and to some extent CCR2, affects some of the inflammatory response to joint destabilisation. Even though levels of Cd68, a macrophage marker, were not significantly raised at 6 h post DMM in WT animals, there was nonetheless a significant suppression of Cd68 in *Ccl2*^−/−^ and *Ccr2*^*−/−*^ joints at this time point. Cd14, another macrophage marker, was elevated significantly in WT mice and was suppressed in *Ccl2*^*−/−*^ and *Ccr2*^*−/−*^ joints, albeit not significantly. Such changes might have been more evident had we measured joint changes at 3 days post DMM, a time at which macrophage levels typically peak after tissue injury^31^. It might also have been more evident had we performed a fluorescence-activated cell sorting (FACS) type analysis on extracts of synovium/capsule using methods described by others^32^. Nonetheless, our results are consistent with unpublished observations that post-operative synovitis, assessed by histology (following destabilisation or sham surgery), peaks at around 24 h post-surgery (predominantly neutrophilic at this stage) and is largely resolved by 7–10 days.

At 6 h post DMM, we were able to document a >90% reduction in Nos2, Ptgs2, Inhba and arginase 1 in *Ccl2*^−/−^ joints, and a more modest but significant suppression of several other genes including Il6, Mmp3 and Timp1. A similar profile was seen in *Ccr2*^*−/−*^ joints although the data set for these genes was incomplete. Conversely, several inflammatory genes, including Has1/2, Arg2 and Mmp3 showed increased joint expression at 7 days post DMM in the knockout joints. Arg1 has many putative functions; its principal role is in protein excretion through the degradation of arginine in the liver, part of the urea cycle. It is also expressed in several other tissues including macrophages where it in part defines the ‘M2’ phenotype^33^. In macrophages, metabolism of arginine by arginase limits the availability of arginine for nitric oxide synthesis and generates ornithine, which can promote polyamine and proline synthesis. Thus high levels of Arg1 may indicate accumulation of a subset of macrophages principally involved in resolution of inflammation and promotion of repair^34^, ^35^. Interestingly, we observed a reciprocal increase in Arg2 levels in both the knockout mice when Arg1 was suppressed. Unlike Arg1, Arg2 is located principally in mitochondria where it also likely plays a key role in modulating nitric oxide and proline synthesis. It has a more restricted tissue distribution and has no deleterious phenotype upon constitutive deletion^36^. Shifts in cellular metabolism have recently been implicated in changing macrophage phenotype^37^ and it is interesting to speculate that deletion of *Ccl2* or *Ccr2* is leading to a shift in metabolic status of the cells of the joint.

Il6, Mmp3, Inhba, Timp1 and Ptgs2 are all well characterised inflammatory response genes with putative roles in OA pathogenesis. Several of these have been examined in knockout studies *in vivo*. For example, the Ptgs 1/2 double knockout mouse did not have altered chondropathy scores following DMM^5^, whereas IL6 has a conflicting role in disease; both an increase in disease with age has been observed as well as decreased disease in experimental OA (induced by DMM) in IL6 knockout mice^6^, ^7^. Mmp3 knockout mice develop a small increase in disease following surgical destabilisation^4^ indicating a neutral or mildly protective role in the joint. Inhibin A is a TGFb family member and is regulated by injury in the joint and cartilage in an FGF2-dependent manner^14^. The marked suppression of Inhba in *Ccl2*^*−/−*^ and *Ccr2*^−/−^ joints post DMM may be largely due to its role in circulating leucocytes where it has macrophage polarizing as well as other immunoregulatory roles^38^. As inflammation is evidently important for promoting tissue repair responses as well as matrix breakdown, the interpretation of the influence of the CCL2/CCR2 axis on joint inflammation post injury makes disease prediction very challenging.

Irrespective of the balance and type of inflammation present in the joints of *Ccl2*^*−/−*^ and *Ccr2*^−/−^ mice, there was no consistent difference in the chondropathy score between these mice at three different time points post DMM. As we amassed significant numbers of animals in these studies, we were able to determine retrospectively that, given the standard deviation, we were powered to detect a difference of 20% between means. Our failure to do so, makes it unlikely that CCL2 or CCR2 has a clinically relevant role in structural OA development. We also included data obtained from a different laboratory in which very similar results, a small non-significant reduction in disease, were obtained. The studies performed in the Vincent lab were by different operators and took place over a long period. When considering these results together, we found that there was a higher than expected spread of disease scores in WT mice, although this did not appear to be due to drift of disease severity over time or due to differences between operators. The results, nonetheless, highlight the importance of being careful to power *in vivo* studies appropriately to overcome this variation.

Our joint structure results were limited to cartilage degradation scores and excluded detailed bone analysis. Osteophytes were present in both *Ccl2*^*−/−*^ and *Ccr2*^−/−^ joints post DMM (data not shown). In *Ccr2*^−/−^ animals from the Malfait study, there was no significant change in presence or size of osteophyte between knockout and WT groups (data not shown). It would have been valuable to do a quantitative analysis of synovitis in the different genotypes, with immunostaining for specific leucocyte markers. Validated measures of synovitis in murine OA have largely been performed on sagittal joint sections, where the reflection of the synovium can be visualised reliably in the anterior and posterior fossae^32^. Visualisation of the synovium from coronal sessions is less reliable and was not performed.

In agreement with previously published studies, we found that although chondropathy scores were not affected by *Ccr2* deletion, there was a change in the course of pain-related behaviour following DMM. Measuring pain-related behaviour at the joint by incapacitance testing, we were able to demonstrate a 4–5 weeks delay in the onset of pain-related behaviour in both *Ccl2*^−/−^ and *Ccr2*^−/−^ mice. Miller *et al.* previously reported that *Ccr2*^−/−^ mice developed transient mechanical allodynia, but failed to exhibit reduced activity (assessed by LABORAS) at 8 and 16 weeks post DMM injury compared with WT animals^18^. This raises the question as to whether reduced activity might have become apparent had they extended the study beyond this time. Their results are consistent with transient inflammatory changes occurring in the dorsal root ganglia at 8 weeks post DMM, perhaps involving activated macrophages. Taken together with our recent data, that point towards direct induction of pain-sensitising molecules by mechanically injured joint tissues themselves^20^, these results suggest that transient regulation of CCL2/CCR2 in the dorsal root ganglion (and perhaps joint), precedes development of pain possibly by increasing the sensitivity of joint tissues to mechanical injury. Antagonising this pathway may have clinical benefits for OA pain but is unlikely to modify structural disease.

## Contributions

Miotla Zarebska J: Analysis and interpretation of the data; Drafting of the article; Final approval of the article; Collection and assembly of data.

Chanalaris A: Analysis and interpretation of the data; Final approval of the article; Statistical expertise.

Driscoll C: Data analysis and interpretation; Final approval of the article; Collection and assembly of data.

Burleigh A: Final approval of the article; Collection and assembly of data.

Miller R: Conception and design; Analysis and interpretation of the data; Critical revision of the article for important intellectual content; Final approval of the article; Collection and assembly of data.

Malfait A-M: Conception and design; Analysis and interpretation of the data; Critical revision of the article for important intellectual content; Final approval of the article; Collection and assembly of data; Obtaining of funding.

Stott B, PhD: Final approval of the article; Collection and assembly of data; provision of technical expertise.

Vincent TL: Conception and design; Analysis and interpretation of the data; Drafting of the article; Critical revision of the article; Final approval of the article; Obtaining of funding.

## Competing interests

Vincent and Malfait are co-guest editors of special issue “Negative *in vivo* studies in OA”. No other conflicts declared.

## Role of funding source

No other roles.

## Figures and Tables

**Table I:** Fold changes of gene expression at 6 h and 7 days post DMM in WT and *Ccl2*^*−/−*^ and *Ccr2*^*−/−*^ mice over their naïve counterparts. C_t_ values were normalised to the levels of RPS18. Adamts – a disintegrin and metalloproteinase with thrombospondin motif

	6 h						7 Days					
	WT/naïve		*Ccl2*^*−/−*^*/*naïve		*Ccr2*^*−/−*^*/*naïve		WT/naïve		*Ccl2*^*−/−*^*/*naïve		*Ccr2*^*−/−*^*/*naïve	
	FC ± sd	*P* value	FC ± sd	*P* value	FC ± sd	*P* value	FC ± sd	*P* value	FC ± sd	*P* value	FC ± sd	*P* value
Adamts4	3.90 ± 0.46	0.012	3.42 ± 0.54	0.003	1.30 ± 0.22	ns	1.87 ± 0.38	ns	1.42 ± 0.03	0.048	0.49 ± 0.18	ns
Adamts5	1.50 ± 0.22	ns	2.96 ± 0.64	ns	1.33 ± 0.23	ns	1.57 ± 0.37	ns	2.26 ± 0.76	ns	1.15 ± 0.06	ns
Arg1	84.23 ± 7.03	<0.001	1.12 ± 0.69	ns	41.46 ± 6.25	0.008	3.14 ± 1.82	ns	2.37 ± 1.19	ns	9.67 ± 0.57	<0.001
Arg2	1.05 ± 0.01	ns	39.49 ± 10.97	ns	28.73 ± 6.61	0.033	1.15 ± 0.16	ns	46.98 ± 3.46	<0.001	5.70 ± 1.73	ns
Ccl2	131.41 ± 18.94	0.005					20.90 ± 1.12	<0.001				
Ccr2	2.24 ± 0.45	ns	1.70 ± 0.19	ns			1.41 ± 0.45	ns	0.62 ± 0.21	ns		
Cd14	5.16 ± 1.25	0.041	3.66 ± 1.33	ns	9.21 ± 1.67	0.028	1.89 ± 0.88	ns	4.89 ± 1.27	ns	4.46 ± 0.56	0.009
Cd68	2.14 ± 0.09	ns	1.04 ± 0.08	ns	0.26 ± 0.09	ns	1.35 ± 0.30	ns	1.32 ± 0.51	ns	0.39 ± 0.09	ns
Has1	3.73 ± 0.46	0.012	5.43 ± 0.26	<0.001	17.50 ± 1.32	<0.001	0.57 ± 0.50	ns	9.23 ± 0.93	0.003	18.08 ± 1.22	<0.001
Has2	2.04 ± 0.06	0.007	1.86 ± 0.47	ns	2.70 ± 0.97	ns	2.35 ± 0.58	ns	9.39 ± 1.39	0.009	2.09 ± 0.43	ns
Il1a	1.21 ± 0.10	ns	1.23 ± 0.10	ns	5.48 ± 1.40	ns	1.02 ± 0.02	ns	0.64 ± 0.11	ns	2.95 ± 0.99	ns
Il1b	5.17 ± 0.28	<0.001	3.65 ± 0.27	0.004	6.65 ± 1.42	0.037	1.92 ± 0.36	ns	5.65 ± 1.58	ns	12.13 ± 0.80	<0.001
Il1r1	3.65 ± 0.68	0.038	5.05 ± 2.56	ns	3.31 ± 2.03	ns	1.31 ± 0.07	ns	2.62 ± 0.48	ns	3.23 ± 0.87	ns
Il6	16.84 ± 2.89	0.012	4.25 ± 0.64	0.025			0.99 ± 0.58	ns	1.45 ± 0.65	ns		
Inhba	2.73 ± 0.09	0.012	0.05 ± 0.03	ns	1.60 ± 0.26	ns	0.70 ± 0.45	ns	0.52 ± 0.03	ns	1.06 ± 0.30	ns
Mmp13	0.42 ± 0.07	0.004	0.72 ± 0.22	ns	0.67 ± 0.15	ns	0.61 ± 0.41	ns	2.47 ± 0.60	ns	0.75 ± 0.12	ns
Mmp3	6.01 ± 0.00	<0.001	2.16 ± 0.19	0.013	1.21 ± 0.23	ns	2.01 ± 0.01	ns	35.78 ± 3.19	0.001	21.82 ± 2.98	0.005
Nos2	14.73 ± 3.23	0.021	0.50 ± 0.03	ns			8.18 ± 1.28	0.015	0.75 ± 0.27	ns		
Ptges	1.44 ± 0.31	ns	0.76 ± 0.05	ns	1.01 ± 0.00	ns	1.25 ± 0.44	ns	0.85 ± 0.10	ns	1.29 ± 0.07	ns
Ptgs2	14.04 ± 2.44	0.012	0.81 ± 0.05	ns	0.65 ± 0.03	ns	9.04 ± 1.82	0.039	0.46 ± 0.07	ns	1.58 ± 0.29	ns
Timp1	4.90 ± 0.44	0.003	0.70 ± 0.22	ns	4.23 ± 1.40	ns	1.24 ± 0.27	ns	1.44 ± 0.22	ns	3.62 ± 0.95	ns
Tsg6	31.19 ± 0.22	<0.001	33.41 ± 2.94	0.001	53.36 ± 15.99	ns	45.27 ± 2.03	<0.001	46.00 ± 3.15	<0.001	11.40 ± 4.37	ns

**Table II:** Ratio of gene expression changes for the *Ccl2*^*−/−*^ or *Ccr2*^*−/−*^ mice over the changes in the WT mice at 6 h and 7 days post DMM. C_t_ values were normalised to the levels of RPS18

	6 h				7 Days			
	*Ccl2*^*−/−*^*/*WT		*Ccr2*^*−/−*^*/*WT		*Ccl2*^*−/−*^*/*WT		*Ccr2*^*−/−*^*/*WT	
	Ratio ± sd	*P* value	Ratio ± sd	*P* value	Ratio ± sd	*P* value	Ratio ± sd	*P* value
Adamts4	0.88 ± 0.17	ns	0.33 ± 0.07	0.015	0.76 ± 0.16	ns	0.26 ± 0.11	ns
Adamts5	1.98 ± 0.52	ns	0.89 ± 0.20	ns	1.44 ± 0.59	ns	0.73 ± 0.18	ns
Arg1	0.01 ± 0.01	<0.001	0.49 ± 0.09	0.022	0.75 ± 0.59	ns	3.08 ± 1.79	ns
Arg2	37.75 ± 10.50	0.0473	27.47 ± 6.33	0.028	40.99 ± 6.47	<0.001	4.98 ± 1.66	ns
Ccl2								
Ccr2	0.76 ± 0.17	ns			0.44 ± 0.20	ns		
Cd14	0.71 ± 0.31	ns	1.78 ± 0.54	ns	2.59 ± 1.38	ns	2.36 ± 1.13	ns
Cd68	0.49 ± 0.04	0.002	0.12 ± 0.04	<0.001	0.98 ± 0.43	ns	0.29 ± 0.09	ns
Has1	1.46 ± 0.19	ns	4.69 ± 0.67	0.001	16.34 ± 14.52	0.003	32.00 ± 28.34	<0.001
Has2	0.91 ± 0.23	ns	1.33 ± 0.48	ns	3.99 ± 1.15	0.025	0.89 ± 0.29	ns
Il1a	1.02 ± 0.12	ns	4.54 ± 1.22	ns	0.63 ± 0.11	ns	2.90 ± 0.98	ns
Il1b	0.71 ± 0.07	0.04	1.29 ± 0.28	ns	2.94 ± 0.99	ns	6.32 ± 1.27	<0.001
Il1r1	1.38 ± 0.75	ns	0.91 ± 0.58	ns	2.01 ± 0.38	ns	2.47 ± 0.68	ns
Il6	0.25 ± 0.06	0.03			1.46 ± 1.08	ns		
Inhba	0.02 ± 0.01	<0.001	0.59 ± 0.10	0.028	0.75 ± 0.48	ns	1.52 ± 1.06	ns
Mmp13	1.74 ± 0.60	ns	1.60 ± 0.45	ns	4.05 ± 2.91	ns	1.23 ± 0.85	ns
Mmp3	0.36 ± 0.03	<0.001	0.20 ± 0.04	<0.001	17.84 ± 1.59	0.001	10.88 ± 1.49	0.006
Nos2	0.03 ± 0.01	0.027			0.09 ± 0.04	0.013		
Ptges	0.53 ± 0.12	0.036	0.70 ± 0.15	ns	0.68 ± 0.25	ns	1.03 ± 0.36	ns
Ptgs2	0.06 ± 0.01	0.014	0.05 ± 0.01	0.012	0.05 ± 0.01	0.025	0.18 ± 0.05	0.037
Timp1	0.14 ± 0.05	0.003	0.86 ± 0.30	ns	1.16 ± 0.31	ns	2.91 ± 0.99	ns
Tsg6	1.07 ± 0.10	ns	1.71 ± 0.51	ns	1.02 ± 0.08	ns	0.25 ± 0.10	0.005
