# Supplementary material for: CCL2 and CCR2 regulate pain-related behaviour and early gene expression in post-traumatic murine osteoarthritis but contribute little to chondropathy
Source: Osteoarthritis Cartilage. 2017 Mar;25(3):406–12. doi: 10.1016/j.joca.2016.10.008 (PMC5358501; doi:10.1016/j.joca.2016.10.008)
Supplement: Supplementary file 1 [file mmc1.docx]

**Supplementary Table 1. Information on** hydrolysis probe assay (Taqman) that were used in the custom TLDAs and their assay numbers as provided by ThermoFisher Scientific, UK.

| **Gene Symbol** | **Gene name** | **Assay ID** | **Chromo-some** | **Target Exons** | **NCBI Gene Reference** |
| --- | --- | --- | --- | --- | --- |
| **18S** | Eukaryotic 18S rRNA | Hs99999901_s1 | 17 | 1 | NM_0011296.2 |
| **Acan** | aggrecan | Mm00545794_m1 | 7 | 2 | NM_007424.2 |
| **Adam8** | a disintegrin and metallopeptidase domain 8 | Mm00545762_m1 | 7 | 19 | NM_007403.2 |
| **Adam9** | a disintegrin and metallopeptidase domain 9 (meltrin gamma) | Mm01218460_m1 | 8 | 21 | NM_007404.2 |
| **Adamts1** | a disintegrin-like and metallopeptidase (reprolysin type) with thrombospondin type 1 motif, 1 | Mm00477355_m1 | 16 | 1 | NM_009621.4 |
| **Adamts15** | a disintegrin-like and metallopeptidase (reprolysin type) with thrombospondin type 1 motif, 15 | Mm01176187_m1 | 9 | 7 | NM_001024139.1 |
| **Adamts4** | a disintegrin-like and metallopeptidase (reprolysin type) with thrombospondin type 1 motif, 4 | Mm00556068_m1 | 1 | 4 | NM_172845.2 |
| **Adamts5** | a disintegrin-like and metallopeptidase (reprolysin type) with thrombospondin type 1 motif, 5 (aggrecanase-2) | Mm00478620_m1 | 16 | 3 | NM_011782.2 |
| **Ar** | androgen receptor | Mm00442688_m1 | X | 2 | NM_013476.3 |
| **Arg1** | arginase, liver | Mm00475988_m1 | 10 | 1 | NM_007482.3 |
| **Arg2** | arginase type II | Mm00477592_m1 | 12 | 3 | NM_009705.3, |
| **Ccl2** | chemokine (C-C motif) ligand 2 | Mm00441242_m1 | 11 | 1 | NM_011333.3 |
| **Ccl5** | chemokine (C-C motif) ligand 5 | Mm01302428_m1 | 11 | 2 | NM_013653.3 |
| **Ccl7** | chemokine (C-C motif) ligand 7 | Mm00443113_m1 | 11 | 1 | NM_013654.3 |
| **Ccr2** | chemokine (C-C motif) receptor 2 | Mm01216173_m1 | 9 | 1 | NM_009915.2 |
| **Ccr5** | chemokine (C-C motif) receptor 5 | Mm01216171_m1 | 9 | 1 | NM_009917.5 |
| **Cd14** | CD14 antigen | Mm00438094_g1 | 18 | 1 | NM_009841.3 |
| **Cd68** | CD68 antigen | Mm00839636_g1 | 11 | 5 | NM_009853.1 |
| **Col2a1** | collagen, type II, alpha 1 | Mm01309565_m1 | 15 | 8 | NM_031163.3, NM_001113515.2 |
| **Ctgf** | connective tissue growth factor | Mm00515790_g1 | 10 | 2 | NM_010217.2 |
| **Esr1** | estrogen receptor 1 (alpha) | Mm00433149_m1 | 10 | 4 | NM_007956.4 |
| **Esr2** | estrogen receptor 2 (beta) | Mm00599819_m1 | 12 | 4 | NM_010157.3 |
| **F3** | coagulation factor III | Mm00438855_m1 | 3 | 3 | NM_010171.3 |
| **Has1** | hyaluronan synthase1 | Mm00468496_m1 | 17 | 4 | NM_008215.2 |
| **Has2** | hyaluronan synthase 2 | Mm00515089_m1 | 15 | 2 | NM_008216.3 |
| **Il1a** | interleukin 1 alpha | Mm00439620_m1 | 2 | 1 | NM_010554.4 |
| **Il1b** | interleukin 1 beta | Mm01336189_m1 | 2 | 1 | NM_008361.3 |
| **Il1r1** | interleukin 1 receptor, type I | Mm00434237_m1 | 1 | 6 | NM_001123382.1, NM_008362.2 |
| **Il1rl1** | interleukin 1 receptor-like 1 | Mm00516117_m1 | 1 | 3 | NM_001025602.2, NM_010743.2 |
| **Il33** | interleukin 33 | Mm00505403_m1 | 19 | 5 | NM_133775.1, |
| **Il6** | interleukin 6 | Mm99999064_m1 | 5 | N/A | NM_031168.1 |
| **Inhba** | inhibin beta-A | Mm00434339_m1 | 13 | 2 | NM_008380.1 |
| **Mmp13** | matrix metallopeptidase 13 | Mm00439491_m1 | 9 | 4 | NM_008607.1 |
| **Mmp19** | matrix metallopeptidase 19 | Mm00491300_m1 | 10 | 6 | NM_021412.1 |
| **Mmp3** | matrix metallopeptidase 3 | Mm00440295_m1 | 9 | 4 | NM_010809.1 |
| **Mmp8** | matrix metallopeptidase 8 | Mm00439509_m1 | 9 | 1 | NM_008611.4 |
| **Nos2** | nitric oxide synthase 2, inducible | Mm01309898_m1 | 11 | 26 | NM_010927.3 |
| **Pdpn** | podoplanin | Mm00494716_m1 | 4 | 1 | NM_010329.2 |
| **Ptges** | prostaglandin E synthase | Mm00452105_m1 | 2 | 2 | NM_022415.2 |
| **Ptgs2** | prostaglandin-endoperoxide synthase 2 | Mm01307329_m1 | 1 | 1 | NM_011198.3 |
| **Saa3** | serum amyloid A 3 | Mm00441203_m1 | 7 | 1 | NM_011315.3 |
| **Serpina1a** | serine (or cysteine) peptidase inhibitor, clade A, member 1a | Mm02748447_g1 | 12 | 15 | NM_009243.3 |
| **Sfrp2** | secreted frizzled-related protein 2 | Mm01213947_m1 | 3 | 2 | NM_009144.2 |
| **Timp1** | tissue inhibitor of metalloproteinase 1 | Mm00441818_m1 | X | 2 | NM_001044384.1 |
| **Tnfaip6** | tumor necrosis factor alpha induced protein 6 | Mm00493736_m1 | 2 | 5 | NM_009398.2 |
| **Tnfrsf12a** | tumor necrosis factor receptor superfamily, member 12a | Mm00489103_m1 | 17 | 1 | NM_013749.2 |
| **Wisp2** | WNT1 inducible signaling pathway protein 2 | Mm00497471_m1 | 2 | 3 | NM_016873.1 |
| **Wnt16** | wingless-related MMTV integration site 16 | Mm00446420_m1 | 6 | 3 | NM_053116.3 |
